# In Vitro Hepatic Clearance Evaluations of Per- and Polyfluoroalkyl Substances (PFAS) across Multiple Structural Categories

**DOI:** 10.3390/toxics12090672

**Published:** 2024-09-14

**Authors:** David M. Crizer, Julie R. Rice, Marci G. Smeltz, Katelyn S. Lavrich, Krishna Ravindra, John F. Wambaugh, Michael DeVito, Barbara A. Wetmore

**Affiliations:** 1 Division of Translational Toxicology, National Institute of Environmental Health Sciences, Research Triangle Park, NC 27711, USA; david.crizer@nih.gov (D.M.C.); julie.rice@nih.gov (J.R.R.); stocksdale.katelyn@epa.gov (K.S.L.); 2 Center for Public Health and Environmental Assessment, Office of Research and Development, US Environmental Protection Agency, Research Triangle Park, NC 27711, USA; smeltz.marci@epa.gov; 3 Oak Ridge Associated Universities, Oak Ridge, TN 37830, USA; ravindra.krishna@epa.gov; 4 Center for Computational Toxicology and Exposure, Office of Research and Development, US Environmental Protection Agency, Research Triangle Park, NC 27711, USA; wambaugh.john@epa.gov (J.F.W.); devito.michael@epa.gov (M.D.)

**Keywords:** PFAS, toxicokinetics (TK), hepatic clearance, in vitro–in vivo extrapolation (IVIVE), New Approach Methods (NAMs), metabolism, biotransformation

## Abstract

Toxicokinetic (TK) assays and in vitro–in vivo extrapolation (IVIVE) models are New Approach Methods (NAMs) used to translate in vitro points of departure to exposure estimates required to reach equivalent blood concentrations. Per- and polyfluoroalkyl substances (PFAS) are a large chemical class with wide-ranging industrial applications for which only limited toxicity data are available for human health evaluation. To address the lack of TK data, a pooled primary human hepatocyte suspension model was used with targeted liquid chromatography–mass spectrometry to investigate substrate depletion for 54 PFAS. A median value of 4.52 μL/(min x million cells) was observed across those that showed significant clearance, with 35 displaying no substrate depletion. Bayesian modeling propagated uncertainty around clearance values for use in IVIVE models. Structural evaluations showed the fluorotelomer carboxylic acids were the only PFAS carboxylates showing appreciable clearance, and per- and polyfluorosulfonamides were more readily metabolized than other PFAS sulfonates. Biotransformation product prediction, using the chemical transformation simulator, suggested hydrolysis of PFAS sulfonamides to more stable sulfonic acids, which is an important consideration for exposure modeling. This effort greatly expands the PFAS in vitro toxicokinetic dataset, enabling refined TK modeling, in silico tool development, and NAM-based human health evaluations across this important set of emerging contaminants.

## 1. Introduction

Per- and polyfluoroalkyl substances (PFAS) are an important class of chemicals with a broad range of industrial applications, molecular structures, and physicochemical and toxicological properties. The U.S. Environmental Protection Agency’s (USEPA’s) Office of Pollution Prevention and Toxics has defined PFAS as any chemical or mixture that includes R-(CF2) C(F)(R′)R″ as a structural unit where none of the R groups are hydrogen and both the CF2 and CF moieties are saturated. This definition is restricted to a proposed ruling under the Toxic Substances Control Act (TSCA) describing reporting and recordkeeping requirements for manufacturers and importers of PFAS [1]. Querying this definition of PFAS against the CompTox Chemicals Dashboard (https://comptox.epa.gov/dashboard/ (accessed on 25 March 2024)) finds over 9000 chemicals, of which approximately 650 of these are currently in commerce [2]. A challenge is that most of these chemicals have no publicly available toxicity data for use in the development of toxicity values. In response to the large numbers of PFAS in commerce and their potential persistence, bioaccumulation, and toxicity, the USEPA developed the National PFAS Testing Strategy: Identification of Candidate Per- and Polyfluoroalkyl Substances (PFAS) for Testing [3]. Due to the hundreds of PFAS in commerce, traditional toxicity testing is untenable to obtain data for all of these chemicals in a timely manner. Thus, the Strategy focuses on grouping similar PFAS into categories and identifying representative substances in each category for testing and prioritization.

As part of the PFAS strategy, initial efforts employ New Approach Methods (NAMs) to assess the bioactivity of up to 150 PFAS, selected based on structural category groupings and regulatory interest. The NAMs focus on multiple endpoints that cover a broad range of bioactivity including assays examining specific transcription factor activities [4]; panels that identify potential immunotoxicants [5]; developmental neurotoxicants [6]; thyroid targets [7]; and other bioactivity evaluations in progress. The chosen assays were based on adverse effects of select PFAS from in vivo toxicity studies and to cover a broader base of bioactivity due to the structural diversity of PFAS. The structural categorization, combined with the in vitro bioactivity data and available in vivo toxicity data, can form the basis of read-across efforts for PFAS [8].

Included in these NAMs are high throughput toxicokinetic assays (HTTK) and in vitro–in vivo extrapolation (IVIVE) modeling. The HTTK assays and IVIVE models provide a means of estimating the exposures required to attain blood concentrations equivalent to the in vitro bioactivity points of departure (PODs). HTTK assays typically focus on plasma protein binding and hepatic clearance due to their importance in overall toxicokinetic (TK) behavior of chemicals. Information from additional assays evaluating gastrointestinal absorption and transporter activity can also be incorporated into the IVIVE models. Earlier human in vitro TK studies from our laboratories have presented plasma protein binding for 110 PFAS [9,10] and plasma protein binding and hepatic clearance of 73 PFAS [10]. These studies demonstrated that the majority of PFAS are highly bound to plasma proteins, with a median bound of 98.91% (f_up_ = 0.0109) and the 75th percentile of 99.6% (f_up_ = 0.0039). PFAS with 6–10 carbon chains were the most highly bound with carbon chains of 11 or more decreasing in binding. In general, PFAS that were more amenable to liquid chromatography–mass spectrometry (LC/MS) analysis were more highly bound than those that were measurable by gas chromatography–mass spectrometry (GC/MS). Of the 73 evaluated by GC/MS for hepatic clearance, abiotic loss (due to nonmetabolic hydrolysis, volatilization, and/or other processes) was observed for 30 PFAS, while only 11 of these PFAS were metabolically cleared. The present study examines hepatic clearance of 54 PFAS that were amenable to LC/MS, comprised primarily of per- and polyfluoryl carboxylic and sulfonic acids, and those containing halides (non-F), ethers, and other functional groups. This work will describe the trends in hepatic metabolism and special considerations required during the IVIVE modeling of PFAS.

## 2. Materials and Methods

### 2.1. Overview

The rate of hepatic clearance was determined as previously described for a set of PFAS [11]. Analysis was conducted across two laboratories at the Division of Translational Toxicology (DTT) at the National Institute of Environmental Health Sciences (NIEHS) and the Center for Computational Toxicology and Exposure at the USEPA. The PFAS analyzed in each lab can be found in the Supplementary Materials.

### 2.2. PFAS Stock Preparation

PFAS stocks were provided through a US EPA contract (Evotec, Inc., Branford, CT, USA). Neat standards obtained from vendors (reported purities >95%) were solubilized at a target concentration of 30 mM in DMSO; lower concentrations were utilized if solubility issues were noted upon visual inspection. DMSO stocks of all PFAS passed an analytical quality-control (QC) evaluation as described in [12]. In this evaluation, stocks were evaluated to ensure presence by accurate mass confirmation and to ensure stability in DMSO. Chemical identifiers, vendor, purity, and category information are provided in Table S1.

### 2.3. Hepatic Clearance Assay

The hepatic clearance assay was performed at both the DTT and EPA laboratories as described below. The pooled human hepatocyte suspensions used in this work were purchased commercially and meet the ethical and scientific standards described in the Human Ethics Rule (40 CFR 26). The suspensions were prepared from hepatocytes obtained from de-identified donors by a commercial vendor. This vendor operates a donor center that is licensed and inspected by the U.S. Food and Drug Administration (BioIVT; bioivt.com (accessed 11 September 2024)). 

PFAS stock solutions prepared in DMSO were added to prewarmed William’s E medium to make a 2X working stock solution (2 µM). The William’s E medium (Cat # A1217601; Thermo Fisher Scientific, Waltham, MA, USA) was supplemented with a cell-maintenance supplement pack (Thermo Fisher Scientific Cat# CM4000) so that the complete media contained 0.1µM of dexamethasone, 2 mM of GlutaMAX, 15mM of HEPES, penicillin/streptomycin (50 μg/mL), and ITS+ supplement (containing insulin (6.25 mg/mL), transferrin (6.25 mg/mL), selenous acid (6.25 ng/mL), bovine serum albumin (1.25 mg/mL), and linoleic acid (5.35 mg/mL)). The working stock solutions were then transferred to a 96-well polypropylene plate and placed in an incubator at 37 °C with 5% carbon dioxide prior to adding cells. Cryopreserved pooled primary human hepatocyte suspensions (50-donor pool, mixed gender; BioIVT; Westbury, NY, USA) were thawed and transferred to vials containing hepatocyte thawing medium. After centrifuging for 5–10 min at 100× *g*, thawing medium was removed, being careful not to disturb the cell pellet. The primary human hepatocytes were then resuspended in pre-warmed complete Williams E medium to achieve a cell density of ~1.0 × 10^6^ viable cells/mL as determined by Trypan Blue exclusion. Cells were then added to the 96-well incubation plates containing PFAS to achieve a final density of ~0.5 × 10^6^ viable cells/mL and a 1 μM assay concentration (C_test_), then placed in incubator on a shaker at 300 rpm. Triplicate samples were removed from the incubator at time points ranging from 0 to 240 min and quenched with a total crash in 2–3 volumes of ice-cold acetonitrile. Samples were spun at 4000× *g* for 20 min, and supernatants were transferred to new tubes and stored at <−70 °C until analysis. Media-only (no cell) and/or heat-inactivated hepatocytes were included as negative controls for each chemical to evaluate and estimate chemical loss due to nonmetabolic processes (e.g., hydrolysis). Phenacetin was included in all runs as an assay reference compound; EPA also included propranolol as an additional reference compound.

### 2.4. Quantification of Chemical Concentrations by Mass Spectrometry (DTT)

Assay samples were diluted in mobile phase and analyzed using a Thermo Vanquish UPLC system with a Thermo Hypersil Gold aQ C18 column (100 × 2.1 mm, 1.9 mm particle size, Thermo Fisher Scientific Inc.) coupled to a Q Exactive Plus mass spectrometer. Gradient elution was used in the chromatographic separations using water with 20 mM ammonium acetate (mobile phase A) and methanol (mobile phase B), with the following gradient program: 0.5–7 min, 30%–90% B; 7–8 min, 90% B; 8–9 min, 90%–30% B; and 9–10 min, 30% B. The first minute of each injection was diverted to waste to avoid introduction of excessive salts from incubation medium. Analysis was detected in negative ion mode. Samples were quantified against an external, matrix-matched calibration curve. Additional methodologic information is available in Table S2.

### 2.5. Sample Preparation and Quantification of Chemical Concentrations by Ultra-Performance Liquid Chromatography (UPLC) Mass Spectrometry (EPA)

Although initial PFAS analysis at the EPA utilized solid-phase extraction (SPE) for sample clean-up, a subset that exhibited stability and/or reproducibility issues was subsequently successfully analyzed without clean-up. To perform SPE, a positive-pressure manifold was employed along with Oasis WAX µElution plates and Oasis HLB 30 mg plates (Waters, Milford, MA, USA) for PFAS and positive-control extraction, respectively. Briefly, WAX plates were conditioned with 2% ammonia in methanol, methanol, and 1% formic acid in water before the supernatant from the metabolic clearance assay was loaded onto the plate. The sorbent was then washed with 1% formic acid and water. Samples were eluted in 2% ammonia in methanol for analysis. Similarly, HLB plates were conditioned with methanol and water before sample was loaded onto the sorbent. The wells were then washed with 5% methanol in water and water before elution in methanol. For samples not undergoing SPE, the supernatant of the crashed assay sample was diluted in mobile phase prior to analysis. The first 1.5 min of the run was diverted to waste to prevent salt contamination. All samples were then transferred to 96-well polypropylene plates for analysis on a Waters ACQUITY I-Class UPLC and Xevo TQ-S micro (UHPLC-MS/MS) and quantitated against a calibration curve using an established laboratory method [9]. Mass-labeled standards (MPFAC-24ES, Wellington Laboratories, Guelph, ON, Canada; Propranolol-d7, CDN Isotopes, Pointe-Claire, QC, Canada; Table S3) were added prior to instrumental analysis. Additional analytical methodologic information (e.g., transitions, internal standards) is included in Tables S2 and S3.

### 2.6. Hepatic Clearance Data Analysis

Data for each PFAS were plotted separately in a semilog format (log concentration vs. time) with three replicates for each time point [11]. Disappearance of PFAS over time was analyzed using linear regression. Data from linear regression (slope) were used to estimate half-life values for each PFAS, which were then used to calculate intrinsic hepatic clearance (Cl_int_). A *p*-value of 0.05 was used to determine significance (i.e., was the slope significantly different from 0), with any PFAS above that cut-off set to a Cl_int_ of 0. Time-matched negative controls were also evaluated similarly to determine if non-metabolic losses were significant.

### 2.7. Experimental Uncertainty Incorporation Using Bayesian Modeling

Markov Chain Monte Carlo was employed in a Bayesian analysis to estimate uncertainty of the hepatic clearance point measures as previously described, but with some modifications [10]. Briefly, after organizing PFAS-level data into a single file, the relationship between the relevant measurement process parameters was described in a graphic model in JAGS language [13] interfaced through R. For hepatic clearance data, the model describes the PFAS-specific mass spectrometric response factor between analyte peak ratio (to internal standard) and chemical concentration. The assumed prior for whether a systematic decrease in chemical was observed followed a Bernoulli distribution with 50% chance of clearance (*p* = 0.5). The assumed prior for whether the chemical degraded abiotically in vitro followed a Bernoulli distribution with a 5% chance of abiotic degradation. Separate biotic and abiotic components for chemical elimination were estimated, each with a slope following an assumed uniform prior distribution bounded between the zero and the -log (limit of quantitation/C_test_). In scenarios where an external calibration without internal standard was used, the response factor was calculated using the analyte peak alone, introducing additional uncertainty due to the inability to normalize for recovery issues. The model was analyzed jointly by JAGS using five Markov chains. Convergence was identified by requiring the five chains to be consistent, as indicated by the potential scale reduction factor [14] being less than 1.1. The analysis was performed using EPA-developed R package invitroTKstats, available upon request. Bayesian approaches have been previously shown to produce estimates of posterior parameter probabilities in which the median value correlates well with the point estimate (as in Section 2.6). What is gained from the Bayesian approach is that the 95% credible interval characterizes uncertainty in the estimate [15]. This uncertainty can be propagated into HTTK predictions using Monte Carlo simulation [15].

### 2.8. In Vitro–In Vivo Extrapolation (IVIVE) and Administered Equivalent Dose (AED) Estimation

IVIVE was performed as previously described [16] to calculate human steady-state concentrations (C_ss_). Briefly, experimental plasma protein binding data presented elsewhere [9] were transformed to fraction unbound in blood using blood-to-plasma partitioning values. Experimental Cl_int_ values generated here are used in conjunction with relevant liver scalars to scale up to estimate whole liver clearance. These data were combined with hepatic blood flow, nonmetabolic renal clearance, and other inputs to estimate C_ss_ values assuming a 1 mg/kg/day exposure rate. Once C_ss_ values were established, these were used with the PFAS-specific in vitro POD data to calculate the administered equivalent doses (AEDs) using reverse dosimetry. The in vitro PODs were derived using published PFAS NAM data [4,5,6] collated from invitrodb v.4.1, also released in September 2023 on the CompTox Chemicals dashboard (https://comptox.epa.gov/dashboard/; last accessed on 22 February 2024). These data were filtered to censor curves with four or more caution flags triggered during data review, and to include only positive curves, using a hitcall threshold of 0.9 for an active curve. Chemicals with “NA” had no positive curves after filtering. The fifth-percentile AC50 value was used to define the in vitro POD. All relevant inputs, scalars, and calculations are provided in Supplementary Information, as indicated in the results.

### 2.9. Chemical Transformation Simulator (CTS) Predictions

The Chemical Transformation Simulator (https://qed.epa.gov/cts/ (accessed on 22 February 2024)) [17] was employed to predict PFAS transformation products following loss of parent analyte due to either hepatocyte metabolism or abiotic hydrolysis in the aqueous assay media. The PFAS reaction library [18] was used with the reaction pathway simulator to derive both metabolic and environmental transformation outputs using the respective reaction libraries. Outputs obtained included transformation route, synthesis code, percent production, percent accumulation, and exact mass of products. Up to four generations were predicted.

## 3. Results

### 3.1. Overview of PFAS Categorization and In Vitro Hepatic Clearance Findings

Experimental Cl_int_ values were measured for 54 PFAS. The initial selection was designed to survey multiple structural and functional groups to address PFAS TK data gaps, making use of categories described by the OECD in 2021 [19] and subsequently used to inform the PFAS-MAP categories [20], used in developing PFAS ToxPrints [21]. Additional information can be found in Tables S1 and S4. While the OECD categories are anchored to structural characteristics and the presence of specific functional groups, the PFAS-Map groupings are broader, combining PFAS with varied compositions and based on synthesis processes. Table 1 shows the structural category coverage across these two frameworks. Additional information can be found in Tables S1 and S4. Also included in Table 1 is the likelihood that the respective PFAS categories are likely to form the more persistent perfluoroalkyl acids (PFAAs), due to either environmental or biotic transformation [22]. Coverage in this study is sufficient to perform comparisons across per- versus polyfluorinated PFAS, in addition to carbon-fluorine chain length (CF2) differences, amongst others. CF2 is defined as the longest contiguous chain of fluorinated carbons. The clearance data, along with the category mappings for each PFAS, are provided in Table S4. PFAS TK data generated in this effort, along with recent publications [9,10,23], can be found in httk package version (2.3.2).

**Figure 1:**
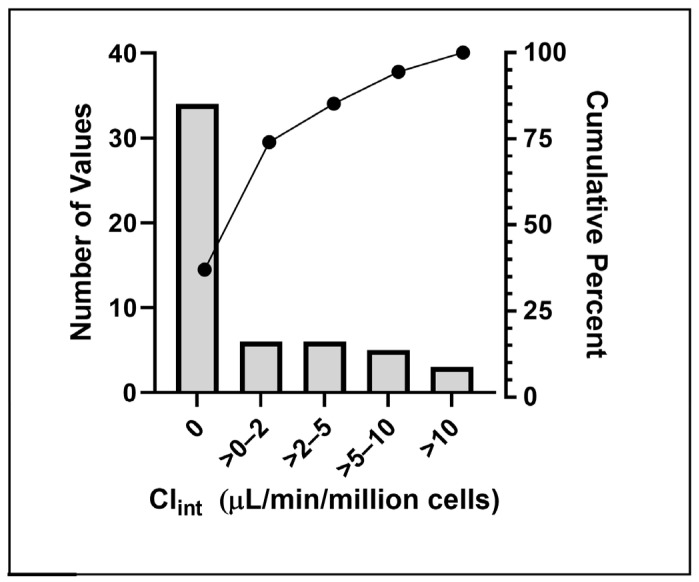
Distribution of Hepatic Intrinsic Clearance across Tested PFAS.

Distribution summary statistics of experimental Cl_int_ rates for the 54 PFAS analyzed indicated these to be fairly stable, with 35 exhibiting no significant hepatic clearance, and 75th and 95th percentile values at 2 and 12 μL/min/million cells, respectively (Figure 1). Only four PFAS had Cl_int_ rates exceeding 10 μL/min/million cells, consistent with a high-clearance metabolized structure.

### 3.2. Category-Based Evaluations of PFAS Metabolic Clearance

**Figure 2:**
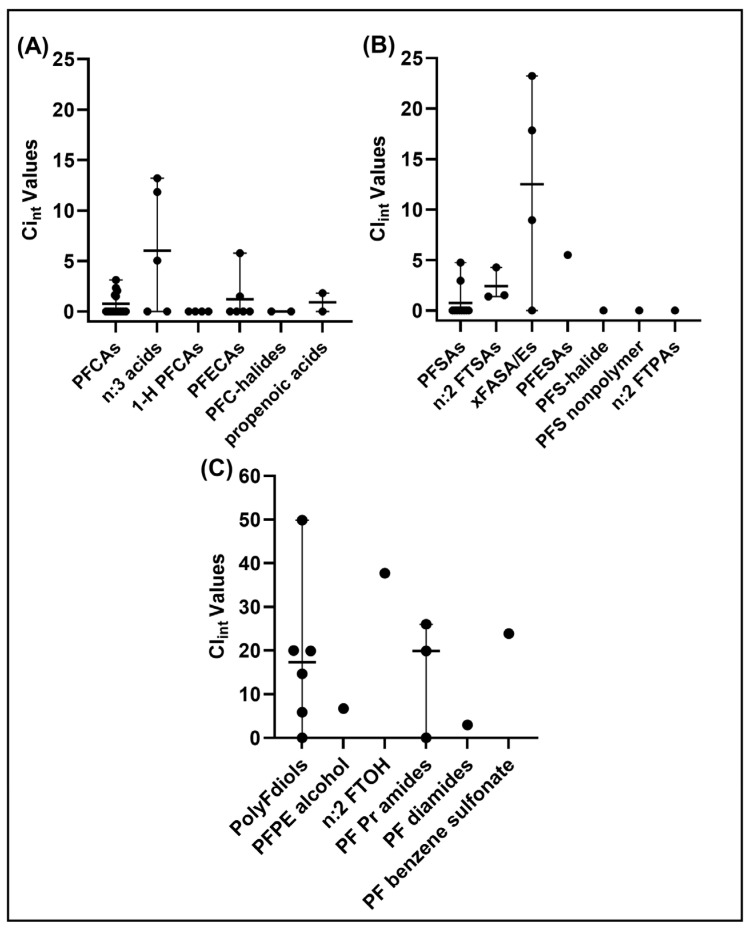
Category-based evaluation of PFAS hepatic intrinsic clearance (Cl_int_). Using groupings described in Table 1, experimental point estimates of Cl_int_ (circles) and associated means (horizontal bars) and ranges (vertical bars) are displayed. (**A**) PF carboxylic and propenoic acids. (**B**) PF sulfonyl groups and FT phosphonic acids (n:2 FTPAs); (**C**) PFAS Cl_int_ from [10]. Abbreviations: PolyF: polyfluorinated; PF: perfluoroalkyl; PE: polyether; FTOH: fluorotelomer alcohol; Pr: primary.

Closer examination of metabolic clearance data for structural-based trends revealed useful observations (Figure 2). Among PFAS categorized structurally as carboxylates, only the n:3 acids, comprising fluorotelomer carboxylic acids, showed any appreciable metabolic clearance. In the PFECA category, a Cl_int_ of 5.78 μL/min/million cells was observed for perfluoro-4-isopropoxybutanoic acid (PFPE-1). For the sulfonate-containing PFAS, the sulfonamides (FASAs) showed the highest metabolic Cl_int_ values, with values exceeding 15 μL/min/million cells for perfluorooctane sulfonamide (PFOSA) and N-Ethylperfluorooctane sulfonamide (NEtFOSA). Abiotic losses were also noted for most of these sulfonamides. Figure 2C provides PFAS Cl_int_ data previously published by the EPA laboratory in Kreutz et al. [10] for a side-by-side comparison. These PFAS were more volatile and required analysis by gas chromatography combined with MS/MS [10]. On average the clearance values were much higher, with 8 of the 13 exhibiting Cl_int_ values exceeding 10.

Table 2 contains chemical-specific Cl_int_ values to enable comparisons across the different chemical categories with the same CF2, as well as comparisons across alkyl acids and sulfonic acids. Representative PFAS were selected from the comprehensive Cl_int_ data summary table provided in Table S4 to provide comparisons across PFAAs, n:3 acids, 1-H PFCAs, PFSAs, and n:2 FTSAs. For the carboxylic acid comparisons, the CF2-matched n:3 acids all exhibited higher Cl_ints_ than their PFAA counterparts, with all but one of the PFAAs shown exhibiting no Cl_int_. For the sulfonic acid comparisons, other than those at the shortest CF2 (i.e., 4), the PFSAs were stable, while the FTSAs exhibited significant but moderate Cl_ints_ of 1.4-1.53. The PFAS at 4 CF2, PFBS, and 4:2 FTS exhibited Cl_ints_ of 47.5 and 4.28, respectively. Additional data are provided in Table S4 for more comparisons.

Using the PFAS described in Table 2, Table 3 brings together the hepatic clearance and plasma protein-binding data to derive C_ss_ values (based on loss of parent compound only) using the generic HTTK model and further estimates AEDs using previously published NAM screening data released in September 2023 (invitrodb v. 4.1), also available on the CompTox Chemicals Dashboard (https://comptox.epa.gov/dashboard/; last accessed on 22 February 2024) [4,5,6]. Experimental f_up_ values range from 0.0004 (PFHpA) to 0.044 (PFPeA); Cl_int_ values range from 0 (12 of the 18) to 15.7 (7:3 FTCA). The C_ss_ predictions range from 4.99 (7:3 FTCA) to 1657.68 μM for PFHpA. When considering CF2, three of the four most potent AEDs belong to the 7 CF2 group, including 9H PFNA and PFOA. The least potent AEDs were noted for polyfluorinated PFAS: shorter-chain sulfonic acids (4:2 FTS, 6:2 FTS) and 5:3 FTCA. Additional data and the underlying calculations are provided in Table S5.

### 3.3. Bayesian Approach–Findings

Across the current and previously published work [10], the Bayesian methods generated more conservative *p*-values for metabolic clearance (Table S6). Of the 21 chemicals with discernable experimental clearance in this study, both analyses agreed that 3-perfluoroheptylpropanoic acid (7:3 FTCA), 2H,2H,3H,3H-perfluorooctanoic acid, and N-methylperfluorooctanesulfonamide were metabolized. Consideration of heat-treated negative controls was used in both approaches to estimate background clearance. In the Bayesian analysis, the decrease observed in these controls was proposed to be large enough to potentially explain the disappearance of the compound in 11 instances. Consideration of such data with experimental estimates in many cases still revealed substrate depletion above and beyond any background clearance that might have been noted (Table S4). Finally, there were seven chemicals where the Bayesian analysis found the data too uncertain to confidently determine if hepatic clearance was observed.

### 3.4. Metabolic Transformation Pathways Using Chemical Transformation Simulator (CTS)

**Figure 3:**
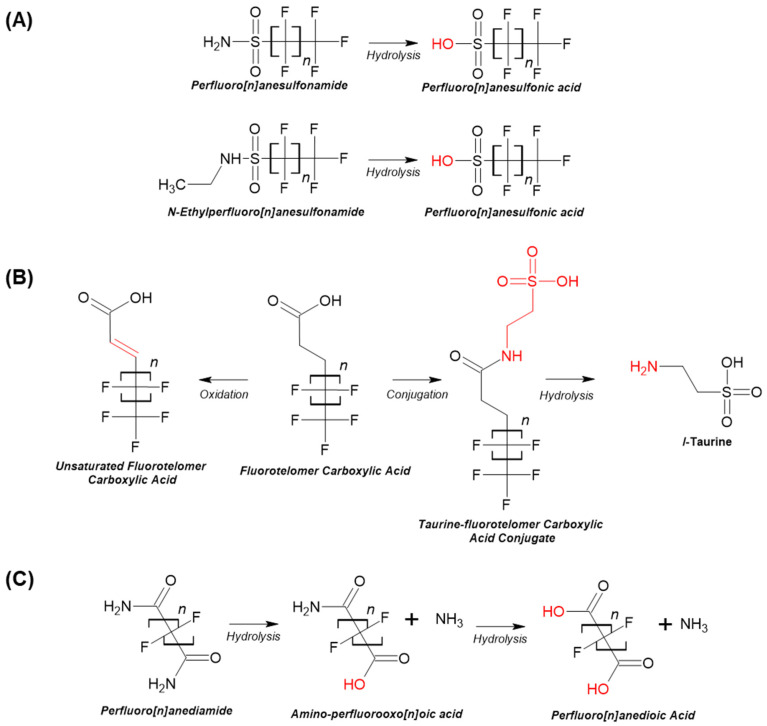
Possible metabolic transformation pathways of PFAS using Chemical Transformation Simulator (CTS). Sulfonamides can transform to sulfonic acids via hydrolysis (**A**), fluorotelomer carboxylic acids can lead to the formation of unsaturated fluorotelomer carboxylic acids and taurine conjugates (**B**), and perfluorinated diamines can become fluorinated dioic acids after two hydrolysis reactions (**C**).

PFAS that were metabolically cleared in vitro were analyzed for theoretical biotransformation products using the PFAS metabolism reaction library within CTS. Three FASAs are likely to undergo hydrolysis to form their respective sulfonic acid: respectively, perfluorooctane sulfonamide will form perfluorooctane sulfonic acid (PFOS); perfluorohexanesulfonamide will form perfluorohexanesulfonic acid (PFHxS); and N-Ethyl perfluorooctane sulfonamide will form PFOS (Figure 3). Polyfluorinated carboxylic or n:3 acids (e.g., 2H,2H,3H,3H-perfluorooctanoic acid or 3:3 fluorotelomer carboxylic acid) are known to undergo oxidation of the resulting fluorotelomer aldehyde to form fluorotelomer carboxylic acid, with a proposed transformation to form the representative carboxylic acid predicted. A full summary of these predicted biotransformation products can be found in Table S7. Figure 3 provides an example of three PFAS compound categories and the potential metabolic transformation pathway they are likely to follow. Evaluation of the environmental reaction library indicated that many of the same PFAS were subject to similar hydrolysis in the environment (Table S8).

## 4. Discussion

Factors driving concern regarding PFAS health effect evaluations are their high bioaccumulative nature combined with low dosages at which human health effects are observed for well-studied legacy PFAAs and PFSAs [24,25,26], contrasted with an almost complete dearth of data for many other PFAS groupings. Until recently, this lack of data encompassed both in vivo and in vitro toxicity and TK evaluations. This effort, in conjunction with recent publications [10,12], provides in vitro TK data, specifically either plasma protein binding or hepatic clearance data, for over 110 PFAS. Brought together, these efforts will serve to inform quantitative IVIVE efforts for these chemicals of emerging concern. This specific contribution adds in vitro hepatic clearance data for 54 PFAS that span multiple categories, including PFAS per- and polyfluorinated carboxylic and sulfonic acids, sulfonamides, and non-fluorine halides; overall, 13 different categories as defined by OECD [19].

TK NAMs readily make use of in vitro assays to evaluate TK properties. These approaches have been designed to complement emerging high-throughput toxicity testing data streams, providing a strategy to rapidly evaluate thousands of data-poor chemicals to identify testing priorities for subsequent studies. To accomplish this, the HTTK package is underpinned by a generic rather than customized model, parameterized with in vitro or in silico hepatic clearance and plasma protein-binding data while using conservative assumptions for other TK inputs in a three-compartment model [27]. When combined with in vitro POD data, in vitro–in vivo concordance evaluations have demonstrated that for most chemicals, this approach provides protective external exposure estimates that are quite useful in NAM-based assessments [28].

In in vitro TK assessments, hepatic clearance is typically evaluated using hepatocyte suspensions due to the commercial availability of high-quality metabolically characterized suspensions, the existence of standardized protocols, their physiologic relevance and ability to recapitulate in vivo metabolism, and the ability to pool multiple individuals to address interindividual variability [29,30]. One of the limitations of this model is the lack of sensitivity in estimating the clearance of low-turnover molecules [31]. It has been estimated that in the typical preparations of 500,000 hepatocytes/mL, the minimum measurable clearance is approximately 2 μL/min/million cells, resulting in an in vivo clearance of approximately 5 mL/min/kg of body weight [32,33]. Notably, the Bayesian methods used in this study, designed to be conservative, provide outputs that are consistent with these earlier evaluations. In reviewing the experimentally derived measures, 63% of the PFAS in this evaluation showed no discernable metabolic clearance, and 75% exhibited Cl_int_ values of 2 μL/min/million cells or lower. This is not necessarily unexpected due to what is known about the stability of legacy PFAAs. There are other hepatocyte culture systems, with varied degrees of complexity and expense, that are promising for evaluating lower turnover xenobiotics: from the use of a hepatocyte relay method [32], an adherent hepatocyte monoculture that can monitor Cl_int_ over 48 hr [34], to more complex co-culture models such as HepatoPac [33] and HμREL systems [35], which extend assay lengths out to 7 days. Should more accurate measurements of the low-clearance PFAS be required, these newer methods may be of value. However, these models are less mature than the suspension approach, requiring further experimental verification and the establishment of best practices regarding the consideration of interindividual variability (single donors are typically used). As currently applied, evaluations in hepatocyte suspensions provide a conservative estimate of hepatic Cl_int_, which is in keeping of a scenario designed to be protective of human health.

The impact of differing PFAS CF2, functional group, and/or fluorination status on PFAS toxicity and fate has already been widely discussed in the literature, with OECD groupings and other PFAS testing selections driven by these observations [8,19,22]. Fluorination status considers whether the carbons on the PFAS backbone are completely occupied (i.e., saturated) with fluorines, known as perfluorinated PFAS, versus those that are not, known as polyfluorinated PFAS. PFAS with a shorter CF2, and those that are polyfluorinated, are recognized as being less stable, so the experimental Cl_int_ data were evaluated for similar trends. Comparisons of perfluorinated versus polyfluorinated PFAS normalized to CF2 (Table 2) indicated that fluorotelomer carboxylic acids (or n:3 acids) exhibited Cl_int_ values ranging from 5–15.7 μL/min/million cells, whereas PFAAs and 1-H PFCAs were stable (i.e., no discernable Cl_int_). This trend was also noted when comparing the PFSAs to the n:2 FTSAs, where significant yet modest Cl_int_ values (i.e., 1.4–4.28 μL/min/million cells) were noted in the latter. These observations provide more empirical support for the polyfluorinated fluorotelomer PFAS, and those PFAS containing sulfonic acids, as exhibiting less stability than the PFAAs. Also, lower CF2 PFAS (e.g., 4–5) typically exhibited higher Cl_int_ values (4.3–11.84 μL/min/million cells) than those 6 or higher. This association of lower CF2 with lower stability is consistent with other reports, including in vivo human biomonitoring findings [19,25]. Experimental evaluations of fluorotelomer carboxylic acid biotransformation indicate oxidation at the site of the unsaturated carbon to form an aldehyde or alternately conjugation to taurine or glutathione (Figure 3B; [36,37]) with no further metabolism. In general, our experimental data and these metabolic evaluations indicate that the unsaturated carbon(s) on these polyfluorinated PFAS is the site(s) of metabolic transformation.

Whereas a loss of the parent compound often indicates the formation of a less toxic hydrophilic metabolite that is more likely to be excreted [38], for PFAS, this is not typically the case. The five PFAS with Cl_int_ values exceeding 8 μL/min/million cells fell in either the n:3 acid/FTCA or the FASA groups—groups with experimental evidence documenting biotransformation to more stable perfluoroalkyl acids [38,39,40]. Particularly for FTCAs and sulfonamides, an evaluation using CTS indicated the formation of more stable alkyl and sulfonic acids (Figure 3). Moreover, evidence exists demonstrating that FASAs and FTOHs undergo abiotic degradation in environmental media, including soils, sediments, and sludge [41,42,43,44]. To ensure adequate protection of human and ecological health, future efforts will need to transition beyond considering only parent compounds toward a more comprehensive incorporation of metabolite formation and environmental fate and transformation information to ensure a real-world PFAS exposure paradigm is adequately captured.

Recent evaluations have indicated that of the two TK parameters, plasma protein binding appears to be more discriminatory than hepatic clearance, due to the wide range of binding observed in earlier efforts [9,10]. Plasma protein binding values across 67 PFAS evaluated in [9] showed a fraction unbound in plasma values spanning 2500-fold, with category-based binding differences driven largely by chain length and functional group presence. Trends analysis of physicochemical properties and plasma protein binding noted the strongest correlations for higher binding with increasing molecular weight and increasing Log P_ow_ values. Due to the lack of metabolism noted for many of the same PFAS tested in the current work, evaluations of any trends regarding clearance and physicochemical properties were not informative. IVIVE calculations that bring these TK data inputs together to derive C_ss_ values provide another useful way to examine these relationships. In the subset displayed in Table 3, there was an interplay between these two inputs, but ultimately, the findings support the importance of plasma protein binding in characterizing the relative persistence of these compounds. What also was clear is that these data were consistent with biomonitoring findings [25] that show that those PFAS with a shorter CF2 (i.e., of 4) have a much shorter half life than those with a CF2 of 7 (e.g., PFOA, PFHxS). Relative comparisons of the C_ss_ values across these groups show that the longer CF2 group (excluding 7:3 FTCA) is 10-to-47-fold higher than the other. Although there may be some questions regarding the overall predictivity of C_ss_ values derived for bioaccumulative chemicals using a generic physiologically based toxicokinetic model, they serve a useful purpose in understanding a relative persistence that, so far, was consistent with other literature reports.

Customization of IVIVE models to reliably evaluate PFAS TK will require specific considerations that are unique to PFAS, as well as other persistent and bioaccumulative chemicals. One of the assumptions employed in the initial application of the generic HTTK model [45] is that xenobiotics reach a steady state rapidly (e.g., within 24 h). This assumption was rooted in earlier pharmacologic research [46,47] that proved reliable when subsequently applied to many non-drug chemicals, as shown in in vitro–in vivo TK data comparisons [16,48]. However, subsequent simulations conducted to evaluate the rigor of this assumption across a larger commercial chemical space showed that, although somewhat appropriate for over 70% of tested chemicals (i.e., 182 of 271 would reach steady-state within 28 days), a subset of 19 chemicals would take over 1000 days to reach a steady state [49]. PFOS and PFOA were two of these 19, with the time to reach steady state was estimated at 6.6 and 3.5 years, respectively. A similar evaluation for PFOS, using the volume of distribution and elimination half-life data obtained from the literature [50,51], employed a one-compartment model that assumed either dynamic or steady-state kinetics [52]. This effort estimated that a dynamic model would take 30 years to reach steady-state values. Any model assuming rapid attainment of a steady state for such persistent chemicals would lead to a significant underestimation that would not be health protective. Having said that, such a steady-state assumption may be acceptable for less stable PFAS, such as the shorter-chain PFAS or polyfluorinated compounds such as the fluorotelomer carboxylic acids, as a PFBS evaluation indicated an estimated half life as low as one month [25]. Overall, these data demonstrate potential value in employing category-based groupings to inform model selection and assumptions.

Further, generic HTTK models typically assume passive chemical excretion (i.e., glomerular filtration) and passive tissue distribution. The unique properties of PFAS indicate that additional aspects of TK warrant consideration beyond what is typically used in a generic HTTK model. For example, the CF2 backbone of PFAS renders them similar to certain endogenous fatty acids that are transporter ligands. Active reuptake via renal transporters on the apical membrane of the proximal tubular cells has been shown to reabsorb PFAS from the urine prior to excretion [53,54,55,56,57]. HTTK overestimates clearance for PFOA and PFOS, leading to predictions for plasma concentrations that were more than 100 times lower than observed [11,49]. As such, incorporation of transporter re-uptake and efflux processes will be important in TK modeling. To date, only a few PFAS have been tested in vitro with a primary focus on renal transporters [58]. PFAS with intermediate chain lengths (between 6 and 10 carbons) show the greatest affinity for transport [59]. Recent [58,60] and any future evaluations of PFAS–transporter interactions will be very informative in refining this aspect of the quantitative IVIVE model.

In summary, this effort, in conjunction with other recent contributions [9,10], provides experimental TK data across several groupings of PFAS beyond the legacy carboxylic and sulfonic acids, greatly expanding our knowledge of PFAS in vitro TK. Although challenges still remain in ensuring all relevant aspects of PFAS TK are adequately characterized and modeled, the category-based assessments of hepatic clearance and plasma protein binding provide useful information for immediate application in read-across approaches and priority–setting.

## Figures and Tables

**Table 1:** Structural category coverage of tested PFAS.

OECD Category ^a,b^	No.	CF2	Potential PFAA Precursor?	PFAS-Map Category
PFCAs	14	lt8 ^c^ = 9; gte8 ^d^ = 5	No ^e^	PFAAs
PFSAs	10	lt8 = 6; gte8 = 4	No	PFAAs
PFECAs	6	lt8 = 4; gte8 = 2	No ^f^	PFAAs
n:3 acids	5	lt8 = 5	Yes	FT PFAA precursors
xFASA-Es	4	lt8 = 1; gte8 = 3	Yes	PFAA precursors; FASA-based PFAA precursors
1H-PFCAs	4	lt8 = 1; gte8 = 3	Yes	PFAA precursors
n:2 FT sulfonic acids	3	lt8 = 1; gte8 = 1	Yes	FT PFAA precursors
PF carbonyl halides	2	lt8 = 2	Yes	Others
PFESAs	1	lt8 = 1	No	PFAAs
PF alkane sulfonyl halides	1	gte8 = 1	Yes	PFAA precursor
PF alkane sulfonyl-based nonpolymers	1	gte8 = 1	Yes	PFAA precursors
n:2 FT phosphonic acids	1	lt8 = 1	Yes	Others

^a^ Of the selected PFAS, two propenoic acids were not categorized in the OECD mappings. ^b^ Abbreviations: carbon-fluorine chain length: CF2; perfluoro (PF) alkyl acids: PFAAs; PF carboxylic acids: PFCAs; <8 CF2: lt8; ≥8 CF2: gte8; PF alkane sulfonic acids: PFSAs; PF ether carboxylic acids (PFECAs); fluorotelomer: FT; PF alkane sulfonyl amides/amido ethanols: xFASA/Es; perfluoroalkane sulfonamide: FASA; PF ether sulfonic acid (PFESA). ^c^ less than 8: lt8. ^d^ greater than or equal to 8: gte8. ^e^ except for perfluoroundecanoic acid. ^f^ except for perfluoro-3-methoxypropanoic acid and perfluoro-3,6-dioxaheptanoic acid.

**Table 2:** Cl_int_ comparisons of per- and polyfluorinated PFAS.

CF2	PFAAs	Cl_int_	n:3 Acids	Cl_int_	1-H PFCAs	Cl_int_	PFSAs	Cl_int_	n:2 FTSAs	Cl_int_
4	PFPeA	0	--		--		PFBS	4.75	4:2 FTS	4.28
5	PFHxA	0	5:3 FTCA	11.84	--	--	--	--	--	--
6	PFHpA	0	6:3 FTCA	5.05	8H-PFOA	0	PFHxS	0	6:2 FTS	1.4
7	PFOA	0	7:3 FTCA	15.7	9H-PFNA	0	PFHpS	0	--	--
8	PFNA	0	--	--	--	--	PFOS	0	8:2 FTS	1.53
9	PFDA	0	--	--	11H-PFUnDA	0	--	--	--	--

**Table 3:** In vitro–in vivo extrapolation to compare C_ss_ and AED estimations.

DTXSID	Compound Name	Mol. Wt. (g/mol)	CF2	OECD Group	f_up_^a,b^	In Vitro Cl_int_ ^a^	C_ss_ (μM) ^a^	AC50 POD (μM) ^c^	AED (mg/kg/day)
DTXSID6062599	PFPeA	263.98	4	PFCAs	0.0440	0	20.78	NA ^d^	NA
DTXSID5030030	PFBS	299.95	4	PFSAs	0.0128	4.75	7.51	6.22	0.83
DTXSID30891564	4:2 FTS	327.98	4	n:2 FTSAs	0.0050	4.28	19.12	NA	NA
DTXSID3031862	PFHxA	313.98	5	PFCAs	0.0068	0	113.04	9.06	0.08
DTXSID20874028	5:3 FTCA	342.01	5	n:3 acids	0.0059	11.84	6.18	6.10	0.99
DTXSID6067331	6:2 FTS	427.98	6	n:2 FTSAs	0.0143	1.4	12.36	11.02	0.89
DTXSID1037303	PFHpA	363.98	6	PFCAs	0.0004	0	1657.68	16.57	0.01
DTXSID70379917	6:3 FTCA	392.01	6	n:3 acids	0.0023	5.05	30.01	5.49	0.18
DTXSID70565479	8H-PFOA	395.98	6	1-H PFCAs	0.0021	0	290.23	10.59	0.04
DTXSID7040150	PFHxS	399.94	6	PFSAs	0.0009	0	670.49	22.38	0.033
DTXSID50226894	9H-PFNA	445.98	7	1-H PFCAs	0.0009	0	601.28	2.47	0.004
DTXSID8059920	PFHpS	449.94	7	PFSAs	0.0006	0	893.98	18.05	0.02
DTXSID90382620	7:3 FTCA	442.01	7	n:3 acids	0.0051	15.7	4.99	0.96	0.19
DTXSID8031865	PFOA	413.97	7	PFCAs	0.0010	0	582.99	8.07	0.01
DTXSID8031863	PFNA	463.97	8	PFCAs	0.0016	0	325.11	14.73	0.05
DTXSID3031864	PFOS	499.94	8	PFSAs	0.0049	0	98.52	8.02	0.08
DTXSID5061954	11H-PFUnDA	545.98	9	1-H PFCAs	0.0015	0	294.69	7.41	0.03
DTXSID3031860	PFDA	513.97	9	PFCAs	0.0027	0	173.91	10.89	0.06

^a^ Fraction unbound in plasma (f_up_); intrinsic clearance (Cl_int_); steady-state concentration (C_ss_); see Section 2 and Table S5 for details on inputs, scalars, and equations. ^b^ f_up_ from [9]. ^c^ 5th percentile AC50 across in vitro bioactivity data in invitrodb v.4.1. See text for details. ^d^ NA, not active; no POD determined during screening.

## Data Availability

Due to the funding of this effort by the US EPA and NIEHS and in compliance with both federal organizations’ Public Access Policy, the accepted, non-formatted version of the accepted manuscript and any associated data files will be made available on Pub Med Central 1 year after acceptance by the journal.

## Supplementary Materials

The following supporting information can be downloaded at: https://www.mdpi.com/article/10.3390/toxics12090672/s1, Supplementary tables: Crizer_etal_2024_PFAS_Hep_Cl_Supp_Tables.xlsx.

## References

[1] USEPA (2021). Toxic Substances Control Act Reporting and Recordkeeping Requirements for Perfluoroalkyl and Polyfluoroalkyl Substances. Fed. Regist..

[2] Williams A.J., Gaines L.G.T., Grulke C.M., Lowe C.N., Sinclair G.F.B., Samano V., Thillainadarajah I., Meyer B., Patiewicz G., Richard A. (2022). Assembly and Curation of Lists of Per- and Polyfluoroalkyl Substances (PFAS) to Support Environmental Science Research. Front. Environ. Sci..

[3] USEPA (2021). National PFAS Testing Strategy: Identification of Candidate Per- and Polyfluoroalkyl Substances (PFAS) for Testing.

[4] Houck K.A., Patlewicz G., Richard A.M., Wiulliams A.J., Shobair M.A., Smeltz M., Clifton M.S., Wemore B., Medvedev A., Makarov S. (2021). Bioactivity profiling of per- and polyfluoroalkyl substances (PFAS) identifies potential toxicity pathways related to molecular structure. Toxicology.

[5] Houck K.A., Paul-Friedman K., Feshuk M., Patlewicz G., Smeltz M., Clifton M.S., Wetmore B.A., Velichko S., Berenyi A., Berg E.L. (2023). Evaluation of 147 Perfluoroalkyl Substances for Immunotoxic and Other (patho)Physiological Activities through Phenotypic Screening of Human Primary Cells.

[6] Carstens K.E., Freudenrich T., Wallace K., Choo S., Carpenter A., Smeltz M., Clifton M.S., Henderson W.M., Richard A.M., Patlewicz G. (2023). Evaluation of Per- and Polyfluoroalkyl Substances (PFAS) In Vitro Toxicity Testing for Developmental Neurotoxicity. Chem. Res. Toxicol..

[7] Degitz S.J., Olker J.H., Denny J.S., Degoey P.P., Hartig P.C., Cardon M.C., Eytcheson S.A., Hasleman J.T., Mayasich S.A., Hornung M.W. (2024). In vitro screening of per- and polyfluorinated substances (PFAS) for interference with seven thyroid hormone system targets across nine assays. Toxicol. Vitr..

[8] Patlewicz G., Richard A.M., Williams A.J., Judson R.S., Thomas R.S. (2022). Towards Reproducible Structure-Based Chemical Categories of PFAS to Inform and Evaluate Toxicity and Toxicokinetic Testing. Comput. Toxicol..

[9] Smeltz M.G., Wambaugh J.F., Wetmore B.A. (2023). Plasma Protein Binding Evaluations of Per- and Polyfluoroalkyl Substances for Category-Based Toxicokinetic Assessment. Chem. Res. Toxicol..

[10] Kreutz A., Clifton M.S., Henderson W.M., Smeltz M.G., Phillips M., Wambaugh J.F., Wetmore B.A. (2023). Category-Based Toxicokinetic Evaluations of Data-Poor Per- and Polyfluoroalkyl Substances (PFAS) using Gas Chromatography Coupled with Mass Spectrometry. Toxics.

[11] Wetmore B.A., Wambaugh J.F., Ferguson S.S., Sochaski M.A., Rotroff D.M., Freeman K., Clewell H.J., Dix D.J., Andersen M.E., Houck K.A. (2012). Integration of dosimetry, exposure, and high-throughput screening data in chemical toxicity assessment. Toxicol. Sci..

[12] Smeltz M.G., Clifton M.S., Henderson W.M., McMillan L., Wetmore B.A. (2023). Targeted Per- and Polyfluoroalkyl substances (PFAS) assessments for high throughput screening: Analytical and testing considerations to inform a PFAS stock quality evaluation framework. Toxicol. Appl. Pharmacol..

[13] Plummer M. JAGS: A program for analysis of Bayesian graphical models using Gibbs sampling. Proceedings of the Third International Workshop on Distributed Statistical Computing (DSC2003).

[14] Gelman A., Rubin D.B. (1992). Inference from iterative simulation using multiple sequences. Stat. Sci..

[15] Wambaugh J.F., Wetmore B.A., Ring C.L., Nicolas C.I., Pearce R.G., Honda G.S., Dinallo R., Angus D., Gilbert J., Sierra T. (2019). Assessing Toxicokinetic Uncertainty and Variability in Risk Prioritization. Toxicol. Sci..

[16] Wetmore B.A., Wambaugh J.F., Allen B., Ferguson S.S., Sochaski M.A., Setzer R.W., Houck K.A., Strope C.L., Cantwell K., Judson R.S. (2015). Incorporating High-Throughput Exposure Predictions With Dosimetry-Adjusted In Vitro Bioactivity to Inform Chemical Toxicity Testing. Toxicol. Sci..

[17] Tebes-Stevens C., Patel J.M., Jones W.J., Weber E.J. (2017). Prediction of Hydrolysis Products of Organic Chemicals under Environmental pH Conditions. Environ. Sci. Technol..

[18] Weber E.J., Tebes-Stevens C., Washington J.W., Gladstone R. (2022). Development of a PFAS reaction library: Identifying plausible transformation pathways in environmental and biological systems. Environ. Sci. Process Impacts.

[19] OECD (2021). Reconciling Terminology of the Universe of Per- and Polyfluoroalkyl Substances: Recommendations and Practical Guidance, in Series on Risk Management.

[20] Su A., Rajan K. (2021). A database framework for rapid screening of structure-function relationships in PFAS chemistry. Sci. Data.

[21] Richard A.M., Lougee R., Adams M., Hidle H., Yang C., Rathman J., Magdziarz T., Bienfait B., Williams A.J. (2023). A New CSRML Structure-Based Fingerprint Method for Profiling and Categorizing Per- and Polyfluoroalkyl Substances (PFAS). Chem. Res. Toxicol..

[22] De Silva A.O., Armitage J.M., Bruton T.A., Dassuncao C., Heiger-Bernays W., Hu X.C., Karrman A., Kelly B., Ng C., Robuck A. (2021). PFAS Exposure Pathways for Humans and Wildlife: A Synthesis of Current Knowledge and Key Gaps in Understanding. Environ. Toxicol. Chem..

[23] Renyer A., Ravindra K., Wetmore B.A., Ford J.L., DeVito M., Hughes M.F., Wehmas L.C., MacMillan D.K. (2023). Dose-Response, Dosimetric, and Metabolic Evaluations of Replacement PFAS Perfluoro-(2,5,8-trimethyl-3,6,9-trioxadodecanoic) acid (HFPO-TeA). Toxics.

[24] Worley R.R., Moore S.M., Tierney B.C., Ye X., Calafat A.M., Campbell S., Woudneh M.B., Fisher J. (2017). Per- and polyfluoroalkyl substances in human serum and urine samples from a residentially exposed community. Environ. Int..

[25] Xu Y., Fletcher T., Pineda D., Lindh C.H., Nilsson C., Glynn A., Vogs C., Norstrom K., Lilja K., Jakobsson K. (2020). Serum Half-Lives for Short- and Long-Chain Perfluoroalkyl Acids after Ceasing Exposure from Drinking Water Contaminated by Firefighting Foam. Environ. Health Perspect..

[26] Zhang Y., Beesoon S., Zhu L., Martin J.W. (2013). Biomonitoring of perfluoroalkyl acids in human urine and estimates of biological half-life. Environ. Sci. Technol..

[27] Pearce R.G., Setzer R.W., Strope C.L., Wambaugh J.F., Sipes N.S. (2017). httk: R Package for High-Throughput Toxicokinetics. J. Stat. Softw..

[28] Paul Friedman K., Gagne M., Loo L., Karamertzanis P., Netzeva T., Sobanski T., Franzosa J.A., Richard A.M., Lougee R.R., Gissi A. (2020). Utility of In Vitro Bioactivity as a Lower Bound Estimate of In Vivo Adverse Effect Levels and in Risk-Based Prioritization. Toxicol. Sci..

[29] Gouliarmou V., Lostia A.M., Coecke S., Bernasconi C., Bessems J., Dorne J.L., Ferguson S., Testai E., Remy U.G., Houston J.B. (2018). Establishing a systematic framework to characterise in vitro methods for human hepatic metabolic clearance. Toxicol. Vitr..

[30] Griffin S.J., Houston J.B. (2004). Comparison of fresh and cryopreserved rat hepatocyte suspensions for the prediction of in vitro intrinsic clearance. Drug Metab. Dispos..

[31] Griffin S.J., Houston J.B. (2005). Prediction of in vitro intrinsic clearance from hepatocytes: Comparison of suspensions and monolayer cultures. Drug Metab. Dispos..

[32] Di L., Obach R.S. (2015). Addressing the challenges of low clearance in drug research. AAPS J..

[33] Chan T.S., Yu H., Moore A., Khetani S.R., Tweedie D. (2019). Meeting the Challenge of Predicting Hepatic Clearance of Compounds Slowly Metabolized by Cytochrome P450 Using a Novel Hepatocyte Model, HepatoPac. Drug Metab. Dispos..

[34] Smith C.M., Nolan C.K., Edwards M.A., Hatfield J.B., Stewart T.W., Ferguson S.S., Lecluyse E.L., Sahi J. (2012). A comprehensive evaluation of metabolic activity and intrinsic clearance in suspensions and monolayer cultures of cryopreserved primary human hepatocytes. J. Pharm. Sci..

[35] Burton R.D., Hieronymus T., Chamem T., Heim D., Anderson S., Zhu X., Hutzler J.M. (2018). Assessment of the Biotransformation of Low-Turnover Drugs in the HmicroREL Human Hepatocyte Coculture Model. Drug Metab. Dispos..

[36] Fasano W.J., Sweeney L.M., Mawn M.P., Nabb D.L., Szostek B., Buck R.C., Gargas M.L. (2009). Kinetics of 8-2 fluorotelomer alcohol and its metabolites, and liver glutathione status following daily oral dosing for 45 days in male and female rats. Chem. Biol. Interact..

[37] Nabb D.L., Szostek B., Himmelstein M.W., Mawn M.P., Gargas M.L., Sweeney L.M., Stadler J.C., Buck R.C., Fasano W.J. (2007). In vitro metabolism of 8-2 fluorotelomer alcohol: Interspecies comparisons and metabolic pathway refinement. Toxicol. Sci..

[38] Issa N.T., Wathieu H., Ojo A., Byers S.W., Dakshanamurthy S. (2017). Drug Metabolism in Preclinical Drug Development: A Survey of the Discovery Process, Toxicology, and Computational Tools. Curr. Drug Metab..

[39] Tomy G.T., Tittlemier S.A., Palace V.P., Budakowski W.R., Braekevelt E., Brinkworth L., Friesen K. (2004). Biotransformation of N-ethyl perfluorooctanesulfonamide by rainbow trout (Onchorhynchus mykiss) liver microsomes. Environ. Sci. Technol..

[40] Zhao S., Wang B., Zhu L., Liang T., Chen M., Yang L., Lv J., Liu L. (2018). Uptake, elimination and biotransformation of N-ethyl perfluorooctane sulfonamide (N-EtFOSA) by the earthworms (Eisenia fetida) after in vivo and in vitro exposure. Environ. Pollut..

[41] D’Agostino L.A., Mabury S.A. (2017). Aerobic biodegradation of 2 fluorotelomer sulfonamide-based aqueous film-forming foam components produces perfluoroalkyl carboxylates. Environ. Toxicol. Chem..

[42] Lange C.C. (2018). Anaerobic biotransformation of N-methyl perfluorobutanesulfonamido ethanol and N-ethyl perfluorooctanesulfonamido ethanol. Environ. Toxicol. Chem..

[43] Liu J., Wang N., Szostek B., Buck R.C., Panciroli P.K., Folsom P.W., Sulecki L.M., Bellin C.A. (2010). 6-2 Fluorotelomer alcohol aerobic biodegradation in soil and mixed bacterial culture. Chemosphere.

[44] Wang N., Szostek B., Buck R.C., Folsom P.W., Sulecki L.M., Gannon J.T. (2009). 8-2 fluorotelomer alcohol aerobic soil biodegradation: Pathways, metabolites, and metabolite yields. Chemosphere.

[45] Rotroff D.M., Wetmore B.A., Dix D.J., Ferguson S.S., Clewell H.J., Houck K.A., Lecluyse E.L., Andersen M.E., Judson R.S., Smith C.M. (2010). Incorporating human dosimetry and exposure into high-throughput in vitro toxicity screening. Toxicol. Sci..

[46] Wilkinson G.R. (1987). Clearance approaches in pharmacology. Pharmacol. Rev..

[47] Gillette J.R., Lawrenson D.E.a.G. (1980). Pharmacokinetic Factors Governing the Steady-State Concentrations of Foreign Chemicals and their Metabolites. Proceedings of the Ciba Foundation Symposium 76—Environmental Chemicals, Enzyme Function and Human Disease.

[48] Wetmore B.A. (2015). Quantitative in vitro-to-in vivo extrapolation in a high-throughput environment. Toxicology.

[49] Wambaugh J.F., Wetmore B.A., Pearce R., Strope C., Goldsmith R., Sluka J.P., Sedykh A., Tropsha A., Bosgra S., Shah I. (2015). Toxicokinetic Triage for Environmental Chemicals. Toxicol. Sci..

[50] Andersen M.E., Butenhoff J.L., Chang S., Farrar D.G., Kennedy Jr G.L., Lau C., Olsen G.W., Seed J., Wallace K.B. (2008). Perfluoroalkyl acids and related chemistries--toxicokinetics and modes of action. Toxicol. Sci..

[51] Olsen G.W., Burris J.M., Ehresman D.J., Froehlich J.W., Seacat A.M., Butenhoff J.L., Zobel L.R. (2007). Half-life of serum elimination of perfluorooctanesulfonate, perfluorohexanesulfonate, and perfluorooctanoate in retired fluorochemical production workers. Environ. Health Perspect..

[52] East A., Dawson D.E., Brady S., Vallero D.A., Tornero-Velez R. (2023). A Scoping Assessment of Implemented Toxicokinetic Models of Per- and Polyfluoro-Alkyl Substances, with a Focus on One-Compartment Models. Toxics.

[53] Nakagawa H., Hirata T., Terada T., Jutabha P., Miura D., Harada K.H., Inoue K., Anzai N., Endou H., Inui K. (2008). Roles of organic anion transporters in the renal excretion of perfluorooctanoic acid. Basic. Clin. Pharmacol. Toxicol..

[54] Nakagawa H., Terada T., Harada K.H., Hitomi T., Inoue K., Inui K., Koizumi A. (2009). Human organic anion transporter hOAT4 is a transporter of perfluorooctanoic acid. Basic. Clin. Pharmacol. Toxicol..

[55] Weaver Y.M., Ehresman D.J., Butenhoff J.L., Hagenbuch B. (2010). Roles of rat renal organic anion transporters in transporting perfluorinated carboxylates with different chain lengths. Toxicol. Sci..

[56] Yang C.H., Glover K.P., Han X. (2010). Characterization of cellular uptake of perfluorooctanoate via organic anion-transporting polypeptide 1A2, organic anion transporter 4, and urate transporter 1 for their potential roles in mediating human renal reabsorption of perfluorocarboxylates. Toxicol. Sci..

[57] Zhao W., Zitzow J.D., Ehresman D.J., Chang S., Butenhoff J.L., Forster J., Hagenbuch B. (2015). Na+/Taurocholate Cotransporting Polypeptide and Apical Sodium-Dependent Bile Acid Transporter Are Involved in the Disposition of Perfluoroalkyl Sulfonates in Humans and Rats. Toxicol. Sci..

[58] Louisse J., Dellafiora L., van den Heuvel J.J.M.W., Rijkers D., Leenders L., Dorne J.C.M., Punt A., Russel F.G.M., Koenderink J.B. (2023). Perfluoroalkyl substances (PFASs) are substrates of the renal human organic anion transporter 4 (OAT4). Arch. Toxicol..

[59] Yang C.-H., Glover K.P., Han X. (2009). Organic anion transporting polypeptide (Oatp) 1a1-mediated perfluorooctanoate transport and evidence for a renal reabsorption mechanism of Oatp1a1 in renal elimination of perfluorocarboxylates in rats. Toxicol. Lett..

[60] Ryu S., Yamaguchi E., Modaresi S.M.S., Agudelo J., Costales C., West M.A., Fischer F., Slitt A.L. (2024). Evaluation of 14 PFAS for permeability and organic anion transporter interactions: Implications for renal clearance in humans. Chemosphere.

